# Therapeutic benefits of an oral vitamin B1 derivative for human T lymphotropic virus type I-associated myelopathy/tropical spastic paraparesis (HAM/TSP)

**DOI:** 10.1186/1741-7015-11-183

**Published:** 2013-08-15

**Authors:** Jun-ichi Kira

**Affiliations:** 1 Department of Neurology, Neurological Institute, Graduate School of Medical Sciences, Kyushu University, Fukuoka, Japan

**Keywords:** Human T lymphotropic virus type I (HTLV-I), HTLV-I-associated myelopathy, Tropical spastic paraparesis, Vitamin B1, Prosultiamine

## Abstract

Prosultiamine, a vitamin B1 derivative, has long been used for beriberi neuropathy and Wernicke’s encephalopathy. Based on the finding that prosultiamine induces apoptosis in human T lymphotropic virus type I (HTLV-I)-infected T cells, Nakamura *et al*. conducted a clinical trial of prosultiamine in patients with HTLV-I-associated myelopathy (HAM)/tropical spastic paraparesis (TSP). In this open-label, single arm study enrolling 24 HAM/TSP patients recently published in *BMC Medicine*, oral prosultiamine (300 mg/day for 12 weeks) was found to be effective by neurological, urological and virological evaluations. Notably, it increased detrusor pressure, bladder capacity and maximum flow rate, and improved detrusor overactivity and detrusor-sphincter dyssynergia. A significant decrease in HTLV-I copy numbers in peripheral blood following the treatment provided a rationale for using the drug. The trial has some limitations, such as the small numbers of participants, the open-label design, the lack of a placebo arm, and the short trial period. Nevertheless, the observation that such a safe, cheap drug may have excellent therapeutic effects on HAM/TSP, a chronic devastating illness occurring mainly in developing countries, provides support for future large-scale randomized controlled trials.

Please see related research: http://www.biomedcentral.com/1741-7015/11/182.

## Background

Prosultiamine (Alinamin®), a well-known thiamine derivative, was first developed by Takeda Pharmaceutical Company in Japan in the 1950s. The drug is a homolog of allithiamine produced by thiol-type vitamin B1 and allicin. Prosultiamine is synthesized by substitution of allyl disulfide with propyl disulfide in the allithiamine structure, to increase its stability in blood and achieve efficient absorption from the gut. Prosultiamine is converted to vitamin B1 after absorption from the gut. The drug thus enables a long-lasting high blood concentration of vitamin B1, resulting in efficient access of vitamin B1 to nervous tissue. Prosultiamine has cured many patients with vitamin B1 deficiency resulting in beriberi neuropathy and Wernicke’s encephalopathy. Prosultiamine might also save patients suffering from another devastating neurologic illness associated with a human retrovirus, namely T lymphotropic virus type I (HTLV-I).

HTLV-I causes not only adult T cell leukemia but also chronic progressive myelopathy, known as HTLV-I-associated myelopathy/tropical spastic paraparesis (HAM/TSP) [1]. HAM/TSP is a devastating neurologic disease causing serious disability, mainly affecting the middle to lower thoracic spinal cord. Patients with HAM/TSP thus present with spastic paraparesis owing to the involvement of the corticospinal tracts. The disease also induces sphincter disturbance, such as dysuria, pollakisuria, urinary retention and constipation, and mild sensory impairment of the lower limbs, such as decreased vibratory sensation and paresthesia. The precise mechanisms underlying HAM/TSP remain to be elucidated, but persistent lymphocytic inflammation exists in the corticospinal tracts and the adjacent white matter of the spinal cord [2]. HTLV-I mainly infects CD4^+^ T cells; such HTLV-I-infected CD4^+^ T cells are autoproliferative and tend to infiltrate central nervous system (CNS) tissue [3]. Some authors have hypothesized the existence of ‘bystander tissue damage’ during the chronic inflammatory process when cytotoxic CD8^+^ T cells continuously kill HTLV-I-infected CD4^+^ T cells infiltrating the CNS [2,4].

In HAM/TSP patients, HTLV-I proviral DNA loads in peripheral blood are markedly increased despite the presence of abundant cytotoxic T cells against HTLV-I [5,6]. Therefore, it is assumed to be beneficial to reduce the number of HTLV-I-infected T cells for treatment of HAM/TSP. Indeed, interferon α (IFNα), a potent drug against the disease, can decrease HTLV-I proviral DNA loads in peripheral blood [7]. Although IFNα is effective, long-term efficacy is modest and adverse effects are relatively frequent and occasionally severe, which makes it difficult to administer the drug for many years. Other immunotherapies, such as corticosteroids [1], plasmapheresis [8], and intermittent high-dose vitamin C [9], exhibit only short-term benefits. Long-term administration of immunosuppressants, such as azathioprine and mizoribine, showed only limited efficacy in a minority of HAM/TSP patients [10]. HAM/TSP is relatively frequently encountered in areas encompassing developing countries, such as equatorial Africa, the Caribbean, Central and South America, the Middle East, and Melanesia [11]. Because curative treatment of HAM/TSP is lacking and no vaccine is available, a low-cost drug with tolerable safety profiles for long-term usage is of paramount importance.

Recently, Nakamura *et al*. [12] discovered that prosultiamine induces apoptosis of HTLV-I-infected T cells; therefore, they conducted a clinical trial of oral prosultiamine in HAM/TSP patients to obtain proof of concept for the drug [13].

### Clinical trial results of prosultiamine in HAM/TSP patients

#### Study design

The study recently published in *BMC Medicine* by Nakamura *et al*. [13] was an open-label, single arm study enrolling 24 patients with HAM/TSP, aged 31 to 80 years. The disease duration ranged from 3 to 51 years, with an average of 20.9 years. Prosultiamine 300 mg was administered orally once daily for 12 weeks. Effects were assessed in three ways, neurological, urological and virological, every 4 weeks. Neurological assessments included the time required for a 10 meter walk, and that required for walking down a flight of stairs, and the modified Ashworth scale (MAS) for spasticity grading of the lower extremities besides full neurological examinations. Detailed urological evaluations were carried out employing the Nocturia Quality of Life (N-QoL) questionnaire and urodynamic studies to measure bladder capacity, detrusor pressure, maximum flow rate, detrusor overactivity, and detrusor-sphincter dyssynergia. Peripheral blood HTLV-I proviral DNA loads were measured by real-time quantitative polymerase chain reaction.

#### Main findings

Prosultiamine improved lower limb spasticity in 80% of the patients (by more than 1 grade for the degree of spasticity on MAS), which almost coincided with improvements in the times required for a 10 meter walk (4.4% to 36.8% decrease in 11 patients) and walking down a flight of stairs (2.3% to 53.2% decrease in 10 patients). Remarkably, both the N-QoL scores and urinary function tests as evaluated by urodynamic studies were significantly improved by the treatment compared with baseline levels; there were increases in detrusor pressure (from 16.8 to 27.5 cm/H_2_O on average) and bladder capacity (from 341.3 to 391.0 ml on average), and maximum flow rate (from 7.5 to 10.2 ml/s on average) together with improvements in detrusor overactivity (68.8% of the patients) and detrusor-sphincter dyssynergia (45.5% of the patients). Importantly, HTLV-I copy numbers in 10^4^ peripheral blood mononuclear cells (PMBCs) decreased significantly after treatment compared with pretreatment levels (from 2,127 to 1,799 on average), with some patients reaching a 30% to 50% decrease. Only three patients complained of mild epigastric discomfort during treatment; otherwise, no side effects were observed.

### Interpretation of the trial findings

The number of participants in the present trial was small and the trial was open-label without a placebo arm; therefore, the current observations should be interpreted with caution and the findings should be confirmed by a large-scale, randomized, double-blind, placebo-controlled study in the future. Nonetheless, the improvement in urodynamic study findings is striking and could provide some proof of concept for the use of prosultiamine in HAM/TSP patients. Although placebo effects in the improved motor performance could not be fully eliminated, the urodynamic study findings support the notion that oral vitamin B1 derivatives have real therapeutic effects on HAM/TSP. The coincidence of the HTLV-I proviral DNA reduction in PBMCs by the drug with therapeutic efficacy may also support such a notion. Such a coincidence was also observed with IFNα [7]. The same authors’ research group previously reported that prosultiamine induces apoptosis of HTLV-I-infected cells, possibly through disruption of the intracellular redox system via reaction of the disulfide moieties in the drug with thiol-containing intracellular molecules [12]. Given that, in two recent studies, a reverse transcriptase inhibitor [14] and a histone deacetylase enzyme inhibitor [15] targeting HTLV-I failed to reduce copy numbers, the significant decrease in HTLV-I copy numbers even after 3 months of administration of prosultiamine in the present study is noteworthy. However, the decrease in HTLV-I proviral DNA loads was modest, albeit significant. Why could such a small decrease in peripheral HTLV-I loads induce measurable clinical benefits? Vitamin B1 easily penetrates into the CNS where it might reduce persistent inflammation via induction of apoptosis of infiltrated HTLV-I-infected cells that express the bcl-2 oncoprotein and are usually resistant to apoptosis [4]. It is critical to investigate whether the elevation of IP-10 and other proinflammatory cytokines in cerebrospinal fluid [16] is downmodulated following treatment with prosultiamine. It is also urgently necessary to clarify if years of prosultiamine administration could further reduce HTLV-I copy numbers. In this case, oral prosultiamine might be classified as a first-line drug for the long-term treatment of HAM/TSP, considering its excellent safety profile.

## Conclusions

Oral prosultiamine could safely alleviate HAM/TSP as evidenced by motor function evaluation and urodynamic studies, coinciding with a modest decrease in HTLV-I copy numbers in PBMCs. The drug appears to be promising, but the long-term benefits of the drug remain to be established by a large-scale, randomized, controlled study. The results of the present study encourage such investigations.

## Competing interests

JK is a consultant for Biogen Idec Japan, and has received honoraria from Bayer Healthcare and funding for a trip from Bayer Healthcare and Biogen Idec Japan. He is funded by a Research Grant for Nervous and Mental Disorders from the Ministry of Health, Labour and Welfare, Japan, and grants from the Japan Science and Technology Agency and the Ministry of Education, Culture, Sports, Science and Technology, Japan.
